# Introducing APOA1 as a key protein in COVID-19 infection: a bioinformatics approach 

**Published:** 2020

**Authors:** Mona Zamanian Azodi, Babak Arjmand, Alireza Zali, Mohammadreza Razzaghi

**Affiliations:** 1 *Proteomics Research Center, Faculty of Paramedical Sciences, Shahid Beheshti University of Medical Sciences, Tehran, Iran*; 2 *Cell Therapy and Regenerative Medicine Research Center, Endocrinology and Metabolism Molecular-Cellular Sciences Institute, Tehran University of Medical Sciences, Tehran, Iran*; 3 *Metabolomics and Genomics Research Center, Endocrinology and Metabolism Molecular-Cellular Sciences Institute, Tehran University of Medical Sciences, Tehran, Iran*; 4 *Functional Neurosurgery Research Center, Faculty of Medicine, Shahid Beheshti University of Medical Sciences, Tehran, Iran*; 5 *Laser Application in Medical Sciences Research Center, Shahid Beheshti University of Medical Sciences, Tehran, Iran*

**Keywords:** urine proteome, COVID-19, protein interaction mapping, biomarkers, biological process

## Abstract

**Aim:**

Introducing possible diagnostic and therapeutic biomarker candidates via the identification of chief dysregulated proteins in COVID-19 patients is the aim of this study.

**Background:**

Molecular studies, especially proteomics, can be considered as suitable approaches for discovering the hidden aspect of the disease.

**Methods:**

Differentially expressed proteins (DEPs) of three patients with demonstrated severe condition (S-COVID-19) were compared to healthy cases by a proteomics study. Cytoscape software and STRING database were used to construct the protein-protein interaction (PPI) network. The central DEPs were identified through topological analysis of the network. ClueGO+CluePedia were applied to find the biological processes related to the central nodes. MCODE molecular complex detection (MCODE) was used to discover protein complexes.

**Results:**

A total of 242 DEPs from among 256 query ones were included in the network. Centrality analysis of the network assigned 16 hub-bottlenecks, nine of which were presented in the highest-scored protein complex. Ten protein complexes were determined. APOA1 was identified as the protein complex seed, and APP, EGF, and C3 were the top hub-bottlenecks of the network. The results specify that up-regulation of C3 and down-regulation of APOA1 in urine play a role in the stiffness in respiration and, accordingly, the severity of COVID-19. Moreover, dysregulation of APP and APOA1 could both contribute to the possible adverse effects of COVID-19 on the nervous system.

**Conclusion:**

The introduced central proteins of the S-COVID-19 interaction network, particularly APOA1, can be considered as diagnostic and therapeutic targets related to the coronavirus disease after being approved with complementary studies.

## Introduction

 COVID-19, a novel severe acute respiratory system syndrome, represents a higher rate of mortality than influenza in the world (1-3). It has been estimated that after the outbreak of influenza (H1N1) in the year 1918, the new coronavirus ranks highest as a global pandemic (4). In March 2020, the World Health Organization (WHO), (https://www.who.int/), announced the outbreak of the new coronavirus as a worldwide pandemic (5). Based on WHO statistics, as of July 25, 2020, the number of confirmed cases globally was 15,581,000, the number of deaths was 635,000, and 216 countries, areas, and territories with the outbreak were recorded. Common symptoms of this disease are dry coughing, fever, fatigue, digestive system problems, difficulty breathing, headache, and muscle soreness (6, 7); the first three indications have been declared by the WHO (https://www.who.int/) as the most common presentations, and the remainder are less frequent ones; however, these signs could vary greatly from individual to individual and from stage to stage (3). In addition, the disease severity could range from mild to severe and the corresponding symptoms also differ (8). As the disease changes from the onset to severe states, the manifestation of symptoms alters, which indicates molecular changes (8). To distinguish the patient’s state prior to symptom development, biomarker analysis could be valuable for predicting the patient’s condition, evaluation, and monitoring (9). For instance, it has been reported that severe COVID-19 (S-COVID-19) cases reveal a high level of cytokine activity termed cytokine storm syndrome (1). These molecule alterations are known as cytokine release syndrome (CRS) and can result in respiratory defects called acute respiratory distress syndrome (ARDS); this stage is very fatal (1, 5). On the other hand, different vital human organs and tissues could be influenced by this kind of virus including the nervous system, cardiovascular system, digestive system, liver, kidney, and blood (10). One of the promising ways to detect these modifications in the related organs in different stages of the disease is to study body fluids such as urine from COVID-19 cases by proteomics, a large-scale protein analysis tool (11). In addition, the application of bioinformatics optimizes the conclusion of these results by exploring the key proteins in terms of interactions which are essential for network strength. Dysregulation in these fundamental proteins of a network could develop into malfunction of the whole interaction system (15). Hence, analysis of the protein-protein interaction network is utilized to recognize the most promising key differentially expressed proteins as potential biomarkers in the network of the severe stage of coronavirus infection. 

## Methods

To conduct protein-protein interaction network analysis in patients with severe COVID-19, 32 samples of healthy people were compared with three COVID-19 patients in severe condition, manifesting fatigue and respiratory dysfunction. All the admitted patients showed symptoms of coughing or fever, and SARS-CoV-2 nucleic acid was positive in their diagnostic test. All the patients also displayed other diseases as comorbidities (11).

In this study, differentially expressed proteins (DEP) with a fold change ≥ 2 and p-value ≤ 0.05 were considered for the network analysis. Cytoscape software 3.8.0 (https://cytoscape.org/) and its applications executed a protein-protein interaction network analysis via the database of STRING v.1.5.1 (http://string-db.org) (12, 13). STRING can perform mapping based on the available sources, i.e. disease name, compound, protein name, and PubMed. The next step was to consider topological properties of the network, including degree (K) and BC by the use of NetworkAnalyzer (14). The nodes with the highest values of degree and betweenness centrality are known as hub-bottlenecks (15). MCODE (Molecular Complex Detection) plug-in then analyzed the network for the potential key condensed areas known as protein complexes. These regions are topologically important due to their multiple interconnections (16). Seed protein in a complex is a node obtained by its node weighting and considering the neighborhood density.

The analysis was performed with these statistical criteria: degree cut off: 2, Haircut: True, Node score cut off: 0.2, K-core: 2, Max. Dept: 100

ClueGO 2.5.7+CluePedia 1.5.7 (17, 18) were assigned for the enrichment analysis of hub-bottlenecks of the severe cases of COVID-19 in comparison with the healthy controls. The candidate biological processes linked to the hub-bottlenecks were explored by the following statistical criteria: Kappa score cut off: 0.5, Max NO of gene per term: 2, Max percentage of genes per term: 3, p-value correction method: Bonferroni step down. 

For the gene ontology grouping, the statistical significance of the terms and groups was calculated by Fisher’s exact test and assigned by a sign indicator in which a dot, two asterisks, and three asterisks are representative of p<0.01, 0.001, p<0.05, and p<0.001, respectively, in the pie chart. 

## Results

In total, the expression of 256 proteins were reported changed in the comparison of healthy samples and patients in severe condition. However, only one protein (HPX) did not meet our statistical criteria in this respect for the network analysis. Among these proteins, 173 showed down-regulation, while the rest indicated up-regulation in the severe condition group. Therefore, most of the proteins are down-regulated in severe statuses of COVID-19 infection. A network of 256 DEPs between the healthy samples and severe condition group was constructed. In this map, 242 proteins as nodes were retrieved with 1298 links. Twenty-two nodes remained as individuals that were not present in the main network. The constructed network was based on the default confidence score cut off =4 with no additional neighbors (figure not shown).

**Figure 1:**
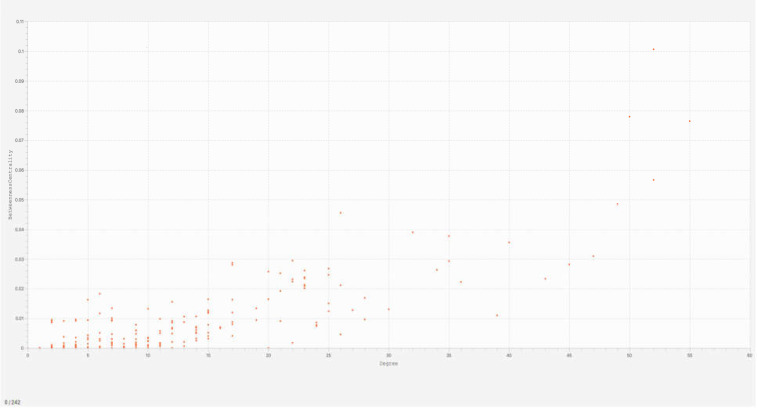
Distribution pattern of network nodes. The x-axis indicates degree value, while the y-axis designates betweenness centrality. The DE proteins are shown as distributed orange dots

**Table 1:** List of central proteins with highest values of degree and betweenness centrality (hub-bottlenecks). Proteins are descending based on degree values. K=Degree, BC=Betweenness Centrality

Row	Gene name	Protein name	K	BC	Regulation
1	APP	Amyloid beta A4 protein	55	0.08	Down
2	C3	Complement C3	52	0.06	Up
3	EGF	Pro-epidermal growth factor	52	0.10	Down
4	APOE	Apolipoprotein E	50	0.08	Down
5	SERPINA1	Alpha-1-antitrypsin	49	0.05	Up
6	APOA1	Apolipoprotein A-I	47	0.03	Down
7	FGA	Fibrinogen alpha chain	45	0.03	Down
8	KNG1	Kininogen-1	43	0.02	Down
9	TTR	Transthyretin	40	0.04	Up
10	HP	Haptoglobin	35	0.03	Up
11	FBN1	Fibrillin-1	35	0.04	Down
12	VTN	Vitronectin	34	0.03	Down
13	CD44	CD44 antigen	32	0.04	Down
14	LAMP1	Lysosome-associated membrane glycoprotein 1	26	0.05	Down
15	B2M	Beta-2-microglobulin	25	0.02	Down
16	HSPG2	Basement membrane-specific heparan sulfate proteoglycan core protein	25	0.03	Down

**Figure 2:**
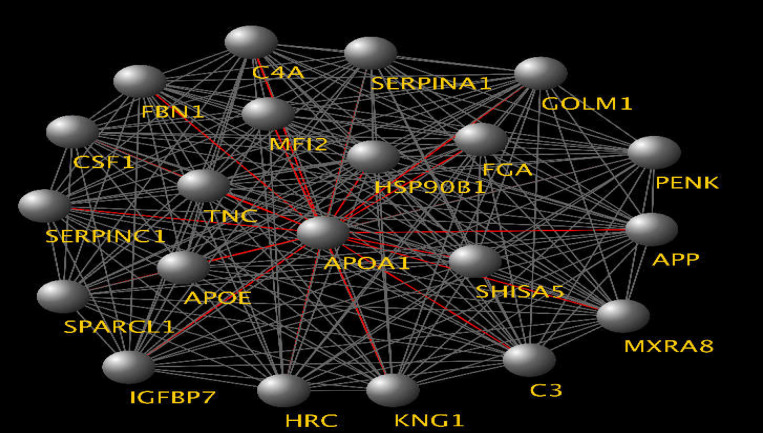
Highest scored cluster of the network of severe patients with COVID-19. APOA1 is the seed protein with the highlighted connections highlighted in red links. Score: 21, NO of nodes: 21 edges: 210

**Figure 3:**
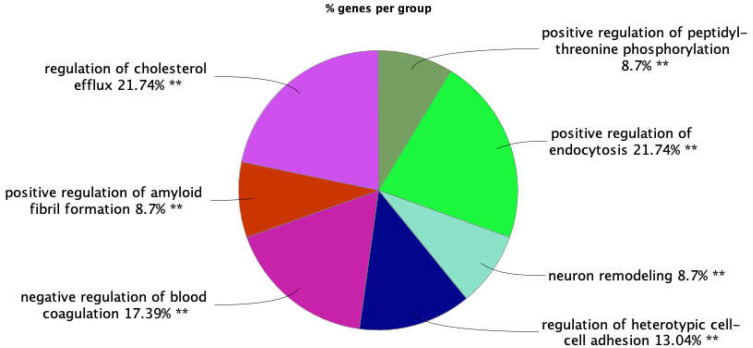
Overview of biological process pie chart of hub-bottlenecks. Kappa score: 0.5, *p*-value: 0.05. Two stars specify the statistically over-significant groups = *p*< 0.001

The centrality study of the DEPs identified some proteins with key topological features. The distribution of these nodes with the key central parameters as degree and betweenness centrality is depicted in Figure 1. The properties of the hub-bottlenecks are listed in Table 1. In Figure 1, the top, right quarter of the rectangle shows the nodes with the highest betweenness centrality and degree values. 

Nodes with high degree and betweenness centrality values have the lowest distribution, indicating a scale free network and the presence of central nodes known as hub-bottlenecks. Table 1 displays 12 down-regulated hub-bottlenecks and 4 up-regulated ones in patients with severe S-COVID-19. APP is the highest ranked hub-bottleneck with a degree of 55 and betweenness centrality of 0.08. 

The MCODE application provided the top interconnected areas as 10 protein clusters. The highest dense protein complex is depicted in Figure 2.

ClueGO and CluePedia were used for the biological process annotation of hub-bottlenecks. The groups of associated terms were assigned with different colors. The groups were prioritized based on the highest percentage of corresponding genes belonging to the groups (see Figure 3).

In the biological process analysis, seven groups were obtained, the two first of which, “regulation of cholesterol efflux” and “positive regulation of endocytosis,” are the most highlighted.

## Discussion

The new coronavirus COVID-19 pandemic is currently an overwhelming crisis globally (19). Therefore, the requirement for discovering molecular therapeutic targets is sensed extensively due to the lethal outcome of the disease and the absence of a precise treatment (20). Different proteomics approaches have been carried out to introduce the potential corresponding dysregulated biomolecules (5, 20-23). Urine is one potential source for proteomics analysis, and was selected for the protein-protein interaction network profiling of COVID-19 patients (3). Differential biomolecules that are key in the network strength have additional importance in disease development (15). In the present investigation, the interaction network of severe coronavirus versus healthy cases was studied. 

A total of 242 proteins were identified in the constructed network by the defined parameters. The centrality analysis explained in Figure 1 revealed that there are central proteins related to the infection, and they are listed in Table 1. Among these sixteen hub-bottlenecks, down-regulation implies domination in the severe condition of COVID-19 network. APOA1 and HP proteins are also reported by another proteomics study of sera, down-regulated, and up-regulated proteins, respectively (20). Amyloid beta A4 protein (APP), complement C3 (C3), pro-epidermal growth factor (EGF), and apolipoprotein E (APOE) are the top four differentially expressed hub-bottlenecks based on the current analysis. The cluster profiling of the network implies that nine hub-bottlenecks are present in the high-scoring cluster of the network. In addition, all the top hub-bottlenecks except EGF are involved in this protein complex. APOA1 is a DE hub-bottleneck that contributes as a seed protein in the highest scored cluster. As previously mentioned, this protein has also been pinpointed as down-regulated in the sera of S-COVID-19 by two other proteomics studies (20, 21). 

Annotation of hub-bottlenecks as biological roles could provide more information about the mechanisms of coronavirus disease. As can be concluded from Figure 3, the groups of terms including “regulation of cholesterol efflux” and “positive regulation of endocytosis” were the most associated biological terms for the hub-bottlenecks; HSPG2, TTR, APOA1, APOE, and EGF belong to the first group, and VTN, B2M, APOE, C3, and EGF are linked to the second group. Moreover, a literature review on the top ranked hub-bottlenecks and the seed protein explains that App (Amyloid beta A4 protein), the novel biomarker of neurodegenerative disease development, is down-regulated in the proteome profile of severe cases of COVID-19. This protein is involved in three groups of biological terms based on our study: “positive regulation of amyloid fibril formation”, “positive regulation of peptidyl-threonine phosphorylation”, and “neuron remodeling.” 

APP as an innate immune particle that shows protective effects as an antiviral agent through catching virus units (24). On the other hand, it has been reported that herpes and influenza virus could induce the trigger of Alzheimer’s disease (AD) (25, 26). It was also mentioned in a recent study that infection with COIVD-19 can promote the development of nervous system diseases, especially AD at a later time (27). Therefore, changes in APP levels in urine samples of severe COVID-19 patients may justify the possible relationship between the virus and APP accumulations and, consequently, its linkage with neurodegenerative diseases.

Complement C3, the next hub-bottleneck, was detected up-regulated in the urine of severe cases. Studies have shown that high levels of C3 indicate severe dysfunctional respiratory illness in COVID-19 patients (28). The protective role of C3 against viral infection is caused by activation of the humoral response (29-31). It has been suggested that the inhibition of C3 activation could be beneficial in the treatment of coronavirus (32, 33), because the high level of inflammatory response could deteriorate the patient’s situation.

The down-regulation of pro-epidermal growth factor (EGF) may indicate the presence of the virus in the kidney and its malfunction based on the fact that the level of EGF is low in the urine of patients with kidney complications (34). Furthermore, kidney problems has previously been associated with severe coronavirus infection (35). It is also known that respiratory illnesses, including coronavirus and influenza, cause the up-regulation of EGFR to facilitate entry into the host cells (36, 37). 

The next novel protein to be evaluated instead of APOE is APOA1, our seed hub-bottleneck. This high-density lipoprotein has been repeatedly reported as decreased in the serum of S-COVID-19 cases (2, 20, 21, 38). In contrast with C3, APOA1 carries anti-inflammatory properties that could assist in the regulation of the immune response (39). The low level of this protein in urine could be induced by corona infection as mentioned above. In addition, aside from the new coronavirus, the correlation between APOA1 with acute respiratory distress syndrome and influenza has been noted by previous studies (38). The down-regulation of APOA1 has been also reported as a part of the HBV pathogenicity mechanism (39). Gene ontology analysis by ClueGO showed that “regulation of cholesterol efflux” and “regulation of heterotypic cell-cell adhesion” are significant associated terms for APOA1. Moreover, brain cholesterol hemostasis is dependent on APOA1 regulation, and its low level could increase the risk of AD (40). Thereby, the feasible post-neurodegenerative mechanism of severe COVID-19 infection could be through the dysregulation of APP and APOA1.

The underlying mechanisms of the pathogenesis and infection of S-COVID-19 could be better described by suggesting APP, EGF, C3, APOE, and APOA1 as the novel chief organizers of the network foundation. Thus, the candidate DE central proteins could be applied as predictors for the severe stage of coronavirus and are worth screening and monitoring for appropriate interventions. It can be suggested that the central dysregulated proteins (APP, EGF, C3, APOE, and APOA1) in the constructed network could be therapeutic targets for clinical approaches for severe COVID-19 conditions. The down-regulated APOA1 in the serum and urine of these patients propose diagnostic features for severity progression. 

## Conflict of interests

The authors declare that they have no conflict of interest.
